# Blood-Derived Products for Tissue Repair/Regeneration

**DOI:** 10.3390/ijms20184581

**Published:** 2019-09-17

**Authors:** Isabel Andia, Nicola Maffulli

**Affiliations:** 1 Regenerative Therapies, Biocruces Bizkaia Health Research Institute, Cruces University Hospital, 48903 Barakaldo, Spain; 2 Department of Musculoskeletal Disorders, University of Salerno School of Medicine and Dentistry, 84084 Salerno, Italy; n.maffulli@qmul.ac.uk; 3 Barts and the London School of Medicine and Dentistry, Queen Mary University of London, London E1 2AD, UK

Medical interest in “blood-derived products for tissue repair/regeneration” has old roots, starting with chronic wounds in the 1980s, and boosted by sports medicine at the beginning of the millennium, when elite athletes treated with platelet rich plasma (PRP) resumed competition earlier than expected. Linking blood-derived products and healing mechanisms is a past milestone that has uncovered distinctive therapeutic approaches for disparate medical conditions. As exemplified in this issue, the transversal nature of blood derived therapies encompasses research that falls in a variety of medical fields: Eye surface in ophthalmology [1], regeneration of bone defects and periodontal tissues in dentistry [2,3,4], osteoarticular pathology, e.g., meniscal repair [5], knee osteoarthritis [6] or radiocarpal osteoarthritis [7]. It also reflects that research in this field encompasses in vitro [4,8,9,10] and in vivo [1,2] experimentation, clinical veterinary [11] and clinical studies [3,5,6,7] representing all the stages of translational research.

We fully acknowledge the explosive growth of research interest in this area, but never have the stakes been higher. After nearly three decades of research, two key questions remain unanswered: What can blood-derived products offer to help understand the intricacies of healing mechanisms? What can we do to foster PRP research and take the benefits it can offer to the varied unmet medical needs? Answering both questions means that we can identify the crucial elements that boost the healing mechanism, and that we can refine formulations to fit with tissue needs in each clinical application.

A holistic approach to deciphering the participation of PRPs in healing mechanisms starts from understanding that the PRP actions are more than those attributed simply to the fact that they contain a vast array of powerful growth factors (GFs). The presence of various GFs in platelets’ alpha granules and their involvement in boosting tissue healing grounded the hypothesis of the clinical use of PRPs, but we should also consider that there is controversy about whether platelet number (concentration factor relative to peripheral blood) and growth factor concentration is paramount in PRP formulations. While traditional approaches of tissue healing research focused on separate GFs, for example recombinant human platelet-derived growth factor (rhPDGF-BB), recombinant human fibroblastic growth factor (rhFGF) or recombinant human epidermal growth factor (rhEGF), have shown some clinical benefits in difficult-to-heal wounds, they provide a limited understanding of healing mechanisms. As postulated by system biology, “networks are more than the sum of the parts”, and this premise helps understanding the difficulties in finding quality parameters able to control efficacies of the different blood derivatives.

In this issue, two reports have focused on the molecular composition of blood-derived products [9,10]. As proposed by Kardos et al. [9], exhaustive scrutiny of the molecular content can provide a guide for developing better formulations tailored to specific tissue/organs’ needs. However, because of the intrinsic complexity of the process of healing, molecular description is not sufficient, and functional in vitro assays can help to differentiate which formulation is more appropriate for a given pathology and/or perhaps different formulations in the different healing stages. Therefore, a priori, there is not a sole effective formulation and different blood derived products may be indicated for the specific tissue needs. Grounded on this idea, Jeyakumar et al. [8] drew parallels between PRP formulation/preparation protocols and healing mechanisms. They evidenced some advantage of hyper-acute autologous serum in enhancing chondrocyte proliferation, while PRP was more useful to stimulate extracellular matrix synthesis and enhance the anabolic/catabolic protein balance in the context of engineered cartilage constructs.

Research could foster new ways of thinking, and can assist in elucidating which blood derivative works better in specific tissues in specific conditions. The molecules released from blood derivatives may transiently form part of the immediate cell microenvironment (local milieu), thereby activating cell function in different tissues/organs. However, different cell phenotypes behave differently in functional terms when exposed to blood derivatives. Moreover, Chellini et al. [12], in a narrative review about skeletal muscle healing, reported that not a single cell phenotype but the collaborative interactions and crosstalk between different cell phenotypes can be modified by PRP. Moreover, platelet poor plasma, PPP, is capturing the attention of researchers in the field of muscle regeneration as it drives cells into the myogenic differentiation pathway and lacks pro-fibrotic factors such as TGF-β1.

Blood derivatives are also interesting as adjuvants, as part of either combination therapies or combination products. Seen in this light, Pomini et al. [2] developed a method to enhance bone formation using platelet-rich fibrin combined with bioOss® (a popular bone filler) and associated to photobiomodulation therapy, in dentistry. Likewise, current research [1] also evaluates the possibility of combining PRP releasate with hyaluronan to treat corneal epithelial defects. In the clinical context, the combination of adipose tissue (more specifically microfat) with PRP is explored as an innovative therapy to treat wrist OA (osteoarthritis) [7].

PRP research must lift its gaze to bigger accomplishments. This will require an improved understanding of repair, which in turn would support treatment strategies, patient selection and the development of precise application procedures. We hope that the identification of biomarkers will help to stratify patients, bringing about more efficient and accurate therapies, better understanding of healing mechanisms and more rational use of PRP therapies and foster the development of new combinational treatments. 
